# C-952-04. Centralized Burn Registry: Elevated Data Integrity

**DOI:** 10.1093/jbcr/irag033.120

**Published:** 2026-04-06

**Authors:** Jennifer M Parks, Taylor Stevens, Farrah Parker, Tracy McDonald, Dustin D Todd

**Affiliations:** HCA Healthcare, Kansas; HCA Healthcare, Liberty Hill, TX; HCA Healthcare, Augusta, Georgia; HCA Healthcare, Nashville, TN; HCA Healthcare - Parallon CDRA, Conway, SC

## Abstract

**Introduction:**

The demand for high-quality healthcare data continues to grow, driving key decisions in patient care and organizational development. Accurate abstraction ensures metric validity and reliability. However, challenges—such as complex data capture, limited staffing, inconsistent abstraction practices, staff turnover, and lack of standardized training—make fulfilling this need difficult. A centralized abstraction model offers structural and operational support to overcome these barriers.

**Methods:**

Our health system includes 11 burn programs nationwide (5 ABA-verified), serving 100 to over 2000 patients annually. Prior to 2024, staffing ratios and inter-rater reliability (IRR) practices varied widely. In some programs, abstraction was shared among multiple staff members. Rising data requirements, including long-term outcomes (LTOs), further strained workflows. In 2024, we implemented a Centralized Data Registry and Abstraction (CDRA) Model. All registrar staff report to a centralized leadership team comprised of a Director, Senior Manager, Operations Manager, Team Lead, and Data Analyst. Goals were set to complete data capture, cross-train staff, improved concurrency, elimination of backlog, standardized education, consistently implement IRR practices, and eliminate non-abstraction responsibilities. Ten programs transitioned in 2024; the final program joined in May 2025.

**Results:**

The CDRA Model standardized staffing to 400:1 and introduced structured onboarding. Additional personnel were hired to meet this standard. Concurrency data collection began January 2025. Initial completion rates were 75% within 60 days and 40% within 30; after six months, rates improved to 100% (60 days) and 87% (30 days). Prior to CDRA, only 3 of 11 programs performed IRR. Post-transition, 10% of all charts are reviewed, yielding a quality score of 97% across 74 data elements.

**Conclusions:**

Centralizing abstraction improved staffing, education, concurrency, and IRR across our burn programs. All 11 centers now meet the 400:1 ratio, conduct IRR on 10% of charts, maintain a 97% data quality score, and close 100% of charts within 60 days of discharge. This system-wide model supports timely, high-quality data collection to drive continuous improvement and better patient outcomes.

**Applicability of Research to Practice:**

The centralized abstraction model provides scalable evidence informed framework to improve data quality, timeliness and consistency. By standardizing staffing ratios, implementing structured onboarding and embedding inter-rater reliability processes, organizations can ensure reliable registry data to inform clinical decision-making, meeting verification requirements and support quality improvement.

**Funding for the study:**

N/A.

